# An Effective Degumming Enzyme from *Bacillus* sp. Y1 and Synergistic Action of Hydrogen Peroxide and Protease on Enzymatic Degumming of Ramie Fibers

**DOI:** 10.1155/2013/212315

**Published:** 2013-03-17

**Authors:** Fenfen Guo, Mouyong Zou, Xuezhi Li, Jian Zhao, Yinbo Qu

**Affiliations:** State Key Laboratory of Microbial Technology, Shandong University, Jinan, Shandong 250100, China

## Abstract

Enzymatic degumming, as an alternative to chemical processing, has attracted wide attention. However, to date, little information about other enzyme components with effective degumming except pectinase has been reported, and there is no report about the effect of bleaching agent (H_2_O_2_) on enzymatic degumming and combining enzymatic degumming and H_2_O_2_ bleaching process. In this study, we found that the crude enzyme of wild-type *Bacillus* sp. Y1 had a powerful and fast degumming ability. Its PGL activity was the highest at pH 9.6–10.0 and 60°C and stable at pH 7–10.5 and 30–50°C, having a wide scope of pH and temperature. Its PGL also had a high H_2_O_2_ tolerance, and the gum loss and brightness of fibers could be significantly improved when H_2_O_2_ was added into it for degumming. The synergistic action was also found between it and H_2_O_2_ on the degumming and bleaching of ramie fibers. All showed that it was very suitable for a joint process of enzymatic degumming and H_2_O_2_ bleaching. It also contained more proteins compared with a control pectinase, and its high protease content was further substantiated as a factor for effective degumming. Protease and pectinase also had a synergistic action on degumming.

## 1. Introduction

Ramie (*Boehmeria nivea*) is widely used in textile industry due to its excellent properties. Its fibers are considered as the longest, strongest, and silkiest in plant fibers and have excellent properties as natural textile material, such as preeminent absorption, quick drying, easy dyeing, shrinkage resistance, good bacteria, mildew, and insect resistance [1]. Moreover, it is also used in ropes, industrial packaging, twines, cordages, canvases, car outfits, and fiber-reinforced composites, among others [2].

However, decorticated ramie fibers contain 20–35% gum, which mainly consists of pectin and hemicellulose. This gum should be removed as much as possible for most applications. Conventional degumming using NaOH solution consumes large amounts of chemicals and energy and causes serious environmental pollution. Therefore, developing new methods for degumming ramie fibers using microorganisms or their enzymes has attracted wide attention, and several reports are available [3–5]. Enzymatic degumming is a gentle reaction, with less damage to fibers and flexible operation, as well as easy processing and quality control, and so on. Hence, it is considered a potential alternative to chemical degumming.

In the enzymatic degumming of ramie fibers, some of the enzymes used in this process, such as pectinase, hemicellulases, and their degumming abilities, have been studied [4]. Pectinase is commonly considered as the key enzyme, and among pectinases, pectate lyase (PGL, E.C 4.2.2.2) is reported to exert more influence on the removal of pectin substances from the fibers than others do [3, 6], whereas high PGL activity in degumming enzymes does not necessarily mean the removal of more pectin and is insufficient for effective degumming because of the complex components of pectin [3, 5]. A suitable enzyme system is necessary for removing pectin in ramie. Parts of literature also showed that xylanase contributes little to the degumming, and lignin-degrading enzymes have little effect on the degumming process attributed to its little lignin content [3, 4, 7]. However, there was no report about the effect of protease with pectinase on enzymatic degumming.

In the textile technology, the bleaching of textile cellulose fabrics by H_2_O_2_ is an established industrial process to improve their performance in further finishing stages. H_2_O_2_ can decolorize, whiten fibers, and remove stains present in it. Much work has been done to elucidate the mechanism of bleaching of cellulose products [8, 9]. Generally, both free radicals and perhydroxyl anions have been suggested as the intermediates in the reactions occurring between cellulosic products and H_2_O_2_. However, the effect of H_2_O_2_ on enzymatic degumming and application of combining enzymatic degumming and H_2_O_2_ bleaching on ramie fibers had not been reported.

In this study, we found that a cellulase-free enzyme from the wild-type *Bacillus* sp. Y1 had a powerful and fast degumming potential on ramie. The mechanism that underlies the effective degumming by it was studied, and its high protease content had been substantiated as a factor for effective degumming. To our knowledge, this is the first report involving the effect of H_2_O_2_ and protease on enzymatic degumming of ramie fibers. The present paper provides a new insight into the enzymatic degumming process and the enzyme component for effective degumming.

## 2. Materials and Methods

### 2.1. Bacterial Strains

All of the strains used in this study were previously isolated from a soil sample collected from bast fiber degumming factory, China, and conserved on nutrient agar slants at 4°C in our laboratory. Crude enzymes from the strains were produced through liquor fermentation to assess their enzymatic degumming potentials. The seed culture medium consists of 10 g/L glucose, 5 g/L peptone, 5 g/L NaCl, 10 g/L K_2_HPO_4_, 0.5 g/L MgSO_4_·7H_2_O, and 5 g/L pectin. The pH of the seed medium was adjusted to 8.0. The strains were incubated on a shaker at 100 rpm and 37°C for 8 h and transferred to the liquor fermentation medium to produce enzyme. The liquid fermentation was conducted in 300 mL Erlenmeyer flasks by taking 50 mL of medium (containing the following: 54 g/L wheat bran; 3 g/L (NH_4_)_2_SO_4_; 2 g/L MgSO_4_·7H_2_O; 1 g/L Na_2_CO_3_; and 1 g/L Tween-80) on a swing shaker (100 rpm) at 34°C for 72 h. The pH of the production medium was adjusted to 8.5 prior to incubation. After fermentation, the cell-free culture supernatant was obtained and used as the crude enzyme for degumming of ramie fibers.

### 2.2. Enzymatic Degumming

Ramie bast fibers were obtained from Yiyang, Hunan Province, China. They were cut into 10 cm pieces and stored in sealed bags at room temperature until use.

Enzymatic degumming of ramie fibers was conducted in 100 mL flasks with enzyme at pH 8.5 (adjusted with glycine-NaOH buffer). The enzyme concentration (based on PGL) was 40 U/g dry ramie, and ramie fiber to liquor ratio was 1 : 13 (w : v). Each contained 2 g of cut ramie fibers and 0.05 g of milled ramie fibers for degumming with crude enzymes and purified proteins, respectively, which wereimmersed in a shaking water bath with a constant temperature (50°C). After the treatment, the fibers were washed and dried at 105°C. To investigate the effect of H_2_O_2_ on enzymatic degumming, 0–48 g/L (based on degumming liquor) of H_2_O_2_ was added into the crude enzyme of strain Y1 for degumming. And fibers treated with 0–48 g/L H_2_O_2_ under the same conditions served as the control.

### 2.3. Analysis of Fiber Properties

The chemical components of ramie fibers were measured according to the China National Standard Method (GB 5889-86). The fiber brightness of ISO was measured on a YQ-Z-48A brightness color tester according to the TAPPI standards [10]. And the brightness of fibers without any treatment was 20% ISO. The gum loss of the fibers (indicated as weight loss) was calculated as follows:

(1) $$\begin{array}{c} \text{Gum}\;\;\text{loss}\;\;\left(\%\right)=\frac{W_{0}-W_{1}}{W_{0}\times W_{2}}\times 100, \end{array}$$

where *W*_0_ and *W*_1_ are the weights of the dry ramie fibers before and after degumming, respectively, *W*_2_ is the gum content of the ramie fibers before degumming. 

### 2.4. Identification of Strain Y1

16S rDNA nucleotide sequences was amplified from chromosomal DNA by PCR using universal oligonucleotide primers and sequenced by BGI, China. The sequences were then compared to the 16S rDNA nucleotide sequences in the GenBank database by BLASTN. The 16S rDNA sequences of isolate Y1 were deposited in the GenBank database with accession number JX910225.

### 2.5. Enzymes

A pectinase was used as the control; commercial protease and mannase were supplied by the Longda Enzymes Company, Shandong, China. Xylanase was fermented from *Penicillium *sp. ZCF57, which was conserved in our laboratory. Their activities were shown in Table 1.

### 2.6. Enzyme Assays

The PGL activity was determined at pH 9.6 by measuring the absorbance of unsaturated bonds in the product at 235 nm. One enzyme unit (U) was defined as the amount of enzyme that produces 1 *μ*mol of unsaturated galacturonic acid per min with a molar extinction coefficient of 4600 [11]. The polygalacturonase (PG, EC 3.2.1.15) activity assay was performed by incubating 0.5 mL of the suitably diluted enzyme with 1 mL of 0.5% pectin (Sigma Chemical Co. type P9135) in 0.05 M glycine-NaOH buffer (pH 9.6) at 55°C for 30 min. One unit (U) was defined as the amount of enzyme required to release 1 *μ*mol of galacturonic acid from polygalacturonic acid per min under the assay conditions [12].

Xylanase (EC 3.2.1.8) and mannase (EC 3.2.1.78) activity were measured at pH 8.5 at 50°C for 30 min according to the procedures of Helianti et al. [13] and Wang et al. [14], respectively. Filter paper (FPase) activity (FPA) of cellulase was measured as described by Liu et al. [15]. The reduced sugars released were analyzed using the DNS assay [16]. One unit (U) of enzyme activity was defined as the amount of enzyme that liberated 1 *μ*mol of reducing sugar per minute under the assay conditions. 

Protease activity was measured by the method of Trisina et al. [17]. One unit (U) was defined as the amount of enzyme that liberates 1 *μ*g of tyrosine per minute.

### 2.7. Characteristics of the Pectinase of the Crude Enzyme from Strain Y1

The optimum pH was determined by measuring the PGL activity in buffered reaction mixtures with pH ranging from 7.0 to 11.0 (0.05 M glycine-NaOH buffer). The optimum temperature for the enzyme activity was determined by assaying enzyme activity at temperatures ranging from 30°C to 70°C at pH 9.6 (0.05 M glycine-NaOH buffer). The pH and temperature stability of the enzyme were studied by exposing the enzyme to buffers with different pH values and incubating the enzyme at various temperatures from 30°C to 70°C. The enzyme samples were taken out at different time intervals, and the residual activity was measured. H_2_O_2_ tolerance of the PGL was investigated by incubating the enzyme in H_2_O_2_ solution with concentrations ranging from 12 g/L to 48 g/L and assaying its residual activity at different incubation intervals. All experiments were carried out in triplicate, and mean values were applied.

### 2.8. Protein Analysis

Sodium dodecyl sulfate polyacrylamide gel electrophoresis (SDS-PAGE) was performed to observe protein composition and determine their molecular masses. The protein bands were directly viewed by staining with Coomassie brilliant blue R250. Molecular weight markers (14.4 kDa to 116 kDa) were used to estimate molecular mass.

### 2.9. Mass Spectrometric Analysis

Matrix-assisted laser desorption/ionization time-of-flight mass spectrometry (MALDI-TOF-MS) was employed to analyze the internal amino acid sequence of the protein bands through in-gel digestion and was sequenced by the Tianjin Biochip Corporation, China.

### 2.10. Purification of Proteins

Concentrated culture filtrate of strain Y1 and control pectinase were loaded onto a Q Fast Flow column (1.6 cm × 20 cm) (GE, Sweden), equilibrated with 20 mM glycine-NaOH buffer (pH 9.26) and NaH_2_PO_4_-Na_2_HPO_4_ buffer (pH 7.5), respectively. The column was eluted with a linear NaCl gradient from 0 M to 1 M in the equilibration buffer at 2 mL/min flow rate, and fractions of 1 mL each were collected. The protease and PGL activity of the fractions were assayed, respectively.

### 2.11. Analysis of Ramie Fibers Treated with Purified Proteins via Scanning Electron Microscopy (SEM)

The ramie fibers were degummed with the purified PGL and the mixture of purified PGL and purified protease, in which the ratio of PGL to protease was consistent with that in the crude enzyme of strain Y1. The ramie fibers with different treatment were scanned via SEM to compare the changes in surface. The ramie fibers were coated with platinum and then studied. Images were taken using a JEOL JSM-6700 SEM (JEOL, Japan).

## 3. Results

### 3.1. Evaluation of the Degumming Potential of the Crude Enzymes from Strains

**Figure 1:**
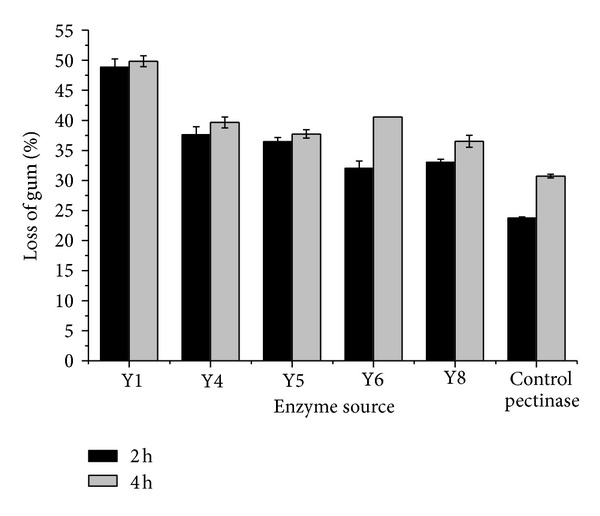
Gum loss of the ramie fibers after degumming with crude enzymes from strains and the control pectinase.

Numerous strains with degumming capabilities were isolated in our laboratory over the past 10 years. Preliminary studies showed that the crude enzymes of strain Y1, Y4, Y5, Y6, and Y8 had good potential in degumming ramie fibers. In this study, the degumming abilities of crude enzymes from strains were further evaluated and compared with a control pectinase solution under the same conditions. The gum loss of ramie fibers after 2 h and 4 h for crude enzymes from strain Y1, Y4, Y5, Y6, and Y8 and the control pectinase were shown in Figure 1. It was apparent that the crude enzyme of strain Y1 had a significantly rapid and powerful degumming potential for ramie fibers. The gum loss of the fibers degumming with the crude enzyme of strain Y1 was 2.1-fold of that with the control pectinase for 2 h. The cellulose content of fibers changed from 71.2% for untreated to 81.0% and 81.2% for degumming after 2 h and 4 h with it, respectively. It further confirmed that the crude enzyme of strain Y1 did not damage ramie fibers. Its activities were detected (Table 1), and it was found that it consisted mainly of pectinase and protease.

### 3.2. Identification of Strain Y1

It was found that the strain Y1 had similar phenotypes characters to *Bacillus *sp., and the 16S rDNA sequence of strain Y1 showed high similarities (>99%) (data not shown) to *Bacillus *sp. further in the similarity search. So, the strain Y1 was identified as *Bacillus *sp.

### 3.3. Characteristics of the Pectinase of the Crude Enzyme from *Bacillus* sp. Y1

**Figure 2:**
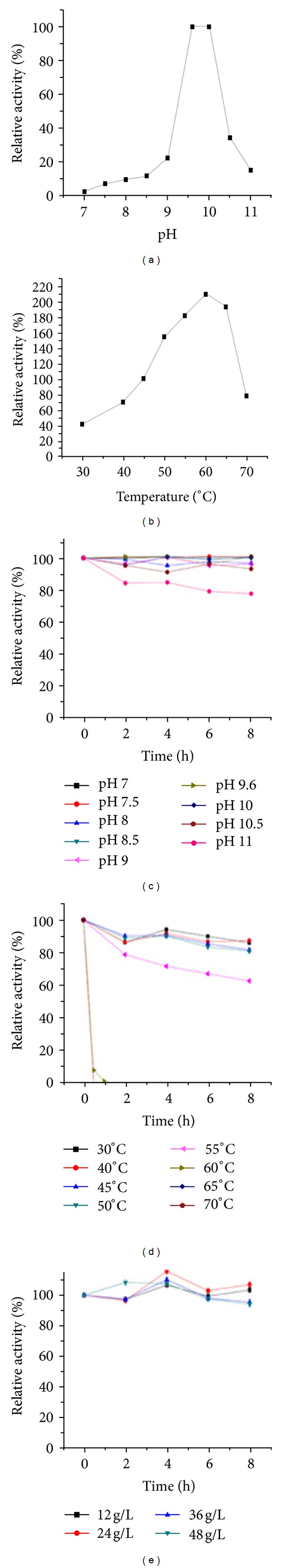
Properties of PGL of the crude enzyme from *Bacillus *sp. Y1: (a) optimum pH; (b) optimum temperature; (c) pH stability; (d) thermal stability; (e) H_2_O_2_ tolerance.

The characteristics of the pectinase of the crude enzyme from *Bacillus *sp. Y1 were also studied to assess its potential in industrial applications. The PGL exhibited a maximum activity at pH 9.6–10.0 (Figure 2(a)) and 60°C (Figure 2(b)) and was stable at pH 7.0–10.5 (Figure 2(c)) and 30–50°C (Figure 2(d)); at least 91% and 81% of its maximum activity were retained within 8 h, respectively. It had wide scope of pH value and temperature. The residual activity was 77% of the initial activity at pH 11.0 and 63% at 55°C after 8 h. The PGL activity was unstable at temperatures up to 60°C, and 92% of its maximum activity was lost at 60°C for 0.5 h. The PGL had a high tolerance for H_2_O_2_, and its activity was even slightly improved in 12–48 g/L H_2_O_2_ solution (Figure 2(e)). Its characteristics indicated that the pectinase of the crude enzyme from *Bacillus *sp. Y1 had a good potential in ramie fiber degumming.

### 3.4. Effect of H_2_O_2_ on Enzymatic Degumming with the Crude Enzyme of *Bacillus* sp. Y1

**Figure 3:**
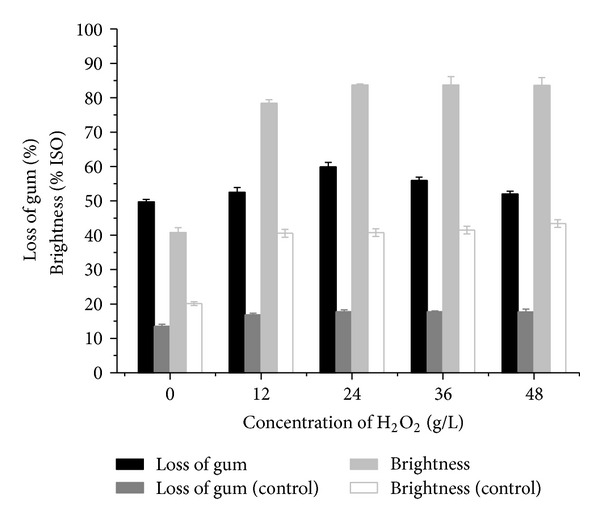
The gum loss and brightness of ramie fibers after degumming with the crude enzyme of *Bacillus *sp. Y1 and different concentration of H_2_O_2_ for 4 h. Control: fibers treated with 0–48 g/L H_2_O_2_ under the same conditions.

The gum loss and brightness of ramie fibers could be significantly improved when a different concentration of H_2_O_2_ was added into the crude enzyme of *Bacillus *sp. Y1 for degumming, especially 24 g/L H_2_O_2_ solution, in which the gum loss and brightness increased by 21% and 1.05-fold, respectively (Figure 3). It was found that the gum loss of the control samples was slightly positively affected by H_2_O_2_ alone. It was also apparently observed that higher gum loss and brightness were achieved by mixture of the crude enzyme of *Bacillus *sp. Y1 and H_2_O_2_ than the amount obtained by using single, which means that there was a synergistic action between the crude enzyme of *Bacillus *sp. Y1 and H_2_O_2_ on the degumming and bleaching of ramie fibers. The crude enzyme from *Bacillus *sp. Y1 was very valuable for combining enzymatic degumming or bioscouring [9] and H_2_O_2_ bleaching of ramie fibers in the industry.

### 3.5. Enzyme Composition Analysis

**Figure 4:**
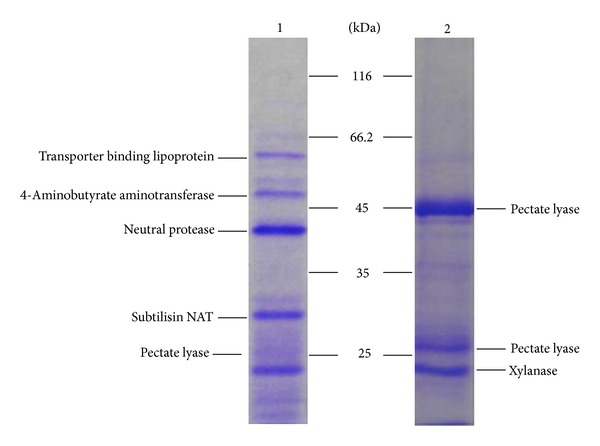
Differences between the enzyme composition of the crude enzyme of *Bacillus *sp. Y1 (1) and control pectinase (2) through SDS-PAGE and MALDI-TOF (MS/MS).

To interpret why the gum loss of fibers for the crude enzyme of* Bacillus *sp. Y1 was much more than the control pectinase at the same conditions (Figure 1), the enzyme compositions of the crude enzyme from *Bacillus *sp. Y1 and the control pectinase were visually compared using SDS-PAGE and analyzed via MALDI-TOF (MS/MS). The main different protein bands in enzyme composition between them were shown in Figure 4, and it was found that more protein types were contained in the crude enzyme of *Bacillus *sp. Y1. For instance, obviously, a much greater amount of protease was present in the crude enzyme of *Bacillus *sp. Y1 compared with the control enzyme (Figure 4). Besides, the high level of the protease activity in the crude enzyme of* Bacillus *sp. Y1 and no protease activity in control pectinase (Table 1) also confirmed this performance.

### 3.6. Identification of the Effect of Protease on Enzymatic Degumming

**Figure 5:**
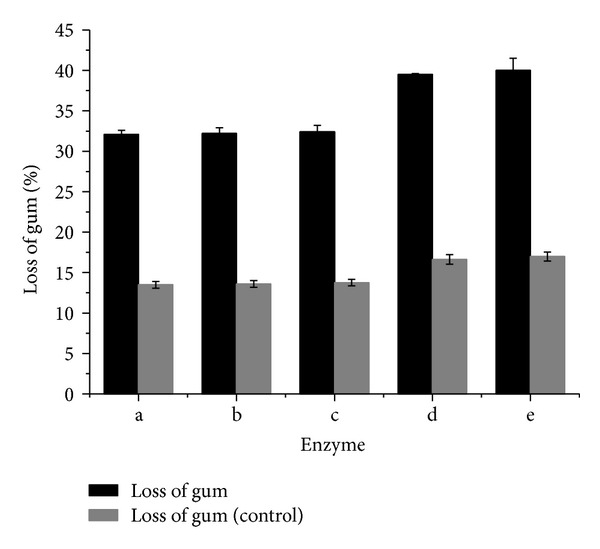
The effect of protease, xylanase, and mannase on the ramie degumming for 4 h: (a) control pectinase; (b) control pectinase + mannase; (c) control pectinase + xylanase; (d) control pectinase + protease; (e) control pectinase + mannase + xylanase + protease. Control: fibers treated with glycine-NaOH buffer, mannase, xylanase, protease and mixture of mannase, xylanase, and protease under the same conditions for (a), (b), (c), (d), and (e), respectively.

To identify whether protease creates the significant difference in gum loss between the crude enzyme of* Bacillus *sp. Y1 and the control pectinase, commercial protease, xylanase, and mannase were added to the control pectinase for degumming with equal amount of each enzyme activity with the crude enzyme of* Bacillus *sp. Y1 in degumming (Figure 5). These three enzymes had no other enzyme activities (Table 1). Fibers treated with glycine-NaOH buffer, mannase, xylanase, protease, and mixture of mannase, xylanase, and protease under the same conditions served as the control, respectively. The results showed that xylanase and mannase had a barely positive effect on degumming of ramie, and protease had a remarkable effect, making the gum loss increased by 26% compared with the control enzyme alone (Figure 5). This increase directly indicated that protease was an important component in the enzymatic degumming of ramie. Figure 5 also showed that protease and pectinase had a synergistic action on the degumming of ramie fibers, due to higher gum loss that could be obtained by mixture of the control pectinase and protease than the amount achieved by using a single enzyme. By measuring activities of PGL in the liquor solution during degumming, it was found that the stability of PGL was not negatively affected when the protease was added into the control pectinase (data not shown).

**Figure 6:**
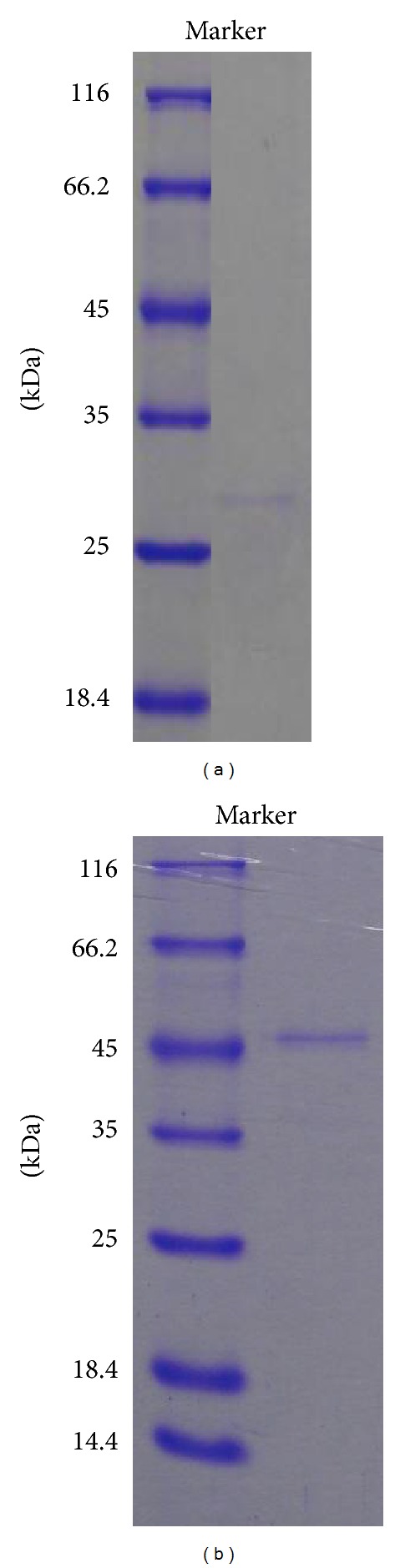
The purified protease (a) and purified PGL (b) separated from the crude enzyme of *Bacillus *sp. Y1 and control pectinase, respectively.

**Figure 7:**
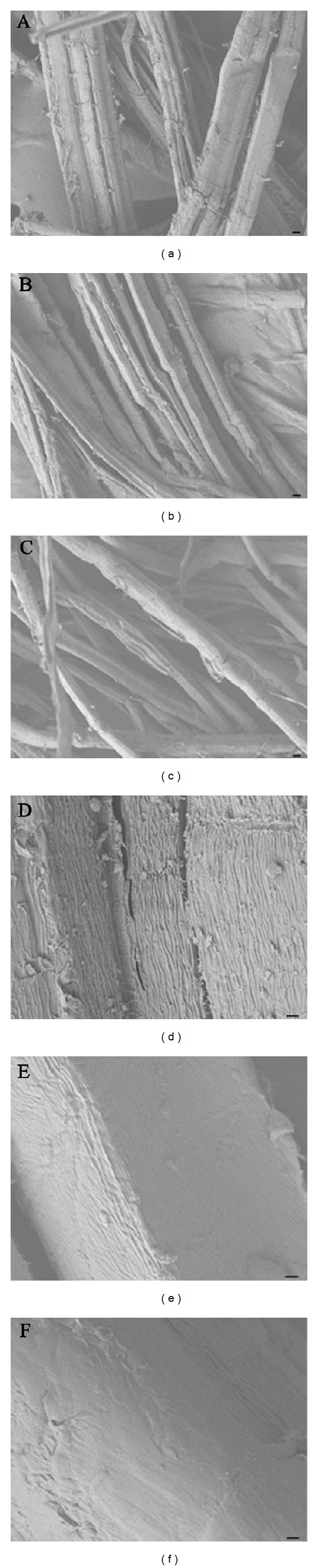
SEM observation of ramie fibers before (a, d) and after treated with the purified PGL (b, e) and the mixture of purified PGL and purified protease (c, f). Scale bars: 10 *μ*m (a, b, and c) and 1 *μ*m (d, e, and f).

 To further verify the effect of protease on ramie degumming, the protease and PGL were purified and then degumming with them. After concentrated culture filtrate of *Bacillus *sp. Y1 was loaded onto Q Fast Flow column, a single band with a molecular weight of 28 kDa could be detected via SDS-PAGE in elution peak (Figure 6(a)). It was the purified protease identified via enzyme determination and MS/MS (Figure 4). A single band with a molecular weight of 45 kDa also could be detected via SDS-PAGE in penetration peak when concentrated control pectinase was loaded onto Q Fast Flow column (Figure 6(b)). It was the purified PGL that wasconfirmed through enzyme determination and MS/MS (Figure 4). When degumming with a mixture of purified PGL and purified protease, in which the ratio of PGL to protease was consistent with that in the crude enzyme of strain Y1, the gum loss of ramie fibers increased by 74% compared with the purified PGL alone for 4 h, and the synergistic action of protease on the degumming of ramie fibers was also observed (data not shown). The surface change of ramie fibers degumming with the purified proteins was shown in Figure 7 by SEM observation.

## 4. Discussion

A number of the genus *Bacillus* and related genera are known to produce extracellular enzymes, which have been applied in ramie fibers industry [3, 5]. Even though some studies about enzymatic degumming had already been done, more effective degumming enzymes are still needed to boost the application of enzymatic degumming technology in the industry [3–5]. In the present study, the wild-type *Bacillus *sp. Y1, which had a cellulase-free enzyme for fast and forceful degumming, was screened. Culture supernatants from *Amycolata* sp. have been reported to be most effective in fiber separation and reducing the gum content of ramie fiber by 30% in 15 h with 240 U PGL/g ramie fibers [4]. However, with the crude enzyme of *Bacillus *sp. Y1 resulted in gum loss up to 48.7% after degumming with shorter time (2 h) and lower PGL dosage (40 U PGL/g ramie fibers). Furthermore, the fiber brightness (40.8% ISO) (Figure 3) was higher than that obtained by degumming with a mixed supernatant of *Bacillus* sp. NT-39 and NT-53 (37.8% ISO) [3].

The PGL activity of *Bacillus *sp. Y1 was highest at pH 9.6–10.0 and 60°C. These are comparable with those of *Bacillus* sp. KSM-P15, having optimal activity around pH 10.5 and 50–55°C [18], and superior to several alkaline pectinases reported by Nasuno and Starr [19], Davé and Vaughn [20], and Magro et al. [21], exhibiting the maximum activity at pH 8.5 and 45°C, and pH 8.0–8.5 and 60°C, pH 8.0 and 30–40°C, respectively. The PGL was stable at pH 7.0–10.5 and 30–50°C, and at least 81% of its maximum activity was retained within 8 h. These also showed that it was suitable for degumming ramie. For example, the wide scope of pH value and temperature made the enzyme of easier use in industrial process because the degumming could be conducted at ambient temperature, and the pH did not need to be adjusted during the degumming process.

The pectinase of the crude enzyme from *Bacillus *sp. Y1 also had a high tolerance for H_2_O_2_, and its activity was even slightly improved in 12–48 g/L H_2_O_2_ solution, which maybe attributed to the action of free radicals and perhydroxyl anions from H_2_O_2_ [9]. What is more, a higher gum loss and fiber brightness were achieved when H_2_O_2_ was added into the crude enzyme of *Bacillus *sp. Y1 for degumming. The highest fiber brightness (83.7% ISO) was much higher than that when using the crude enzyme of *Bacillus *sp. Y1 (40.8% ISO) and H_2_O_2_ alone (40.8% ISO) [3]. Based on the results, it was suggested that the crude enzyme of *Bacillus *sp. Y1 and H_2_O_2_ had a synergistic action on the degumming and bleaching of ramie fibers (Figure 3). Zheng et al. reported that enzymatic degumming could result in the removal of part chromophoric material linking gum components [3]. Ibrahim et al. also reported that the pectinase has the ability to hydrolyze and transform the water-insoluble polygalacturonic acid into water-soluble oligomers, thereby enhancing the release of other hydrophobic noncellulosic impurities away from the wall, as well as facilitating and increasing the extent of H_2_O_2_-bleaching via creation of more available active-surface area for further modification, and H_2_O_2_ further removes the colored substances and stains present in the fibers increasing the contact area of pectinase and pectic substance to promote the enzyme action [9]. So, this crude enzyme could be used in combining enzymatic degumming or bioscouring and H_2_O_2_ bleaching. Therefore, this enzyme and this process could not only decrease the process, device, and energy consumption to reduce the production costs but also obtain higher gum loss and fiber brightness. On the basis of these results, the crude enzyme of *Bacillus *sp. Y1 showed great potential in the fiber processing industry.

Few studies have focused on other effective enzyme compositions for fiber degumming except pectinase. Generally, the PGL is known as the key enzyme [3, 6]. However, hemicellulases, for example, xylanase and mannase, do not significantly contribute to ramie degumming [3, 6], with which our results were consistent (Figure 5). In this study, we found that the crude enzyme system of *Bacillus* sp. Y1 had a greatly higher content of protease than that of the control pectinase (Figure 4 and Table 1), and the protease was substantiated to play a much more significant role in degumming ramie (Figure 5). Protease is one of the most important industrial enzymes in sericin (gum) removal from silk yarn [22]. However, few reports on the application of protease in ramie degumming have been published.

Using Kjeldahl determination [23], it was found that the ramie used in the current paper contained 3.3% protein, of which 67% were removed after degumming with the crude enzyme of *Bacillus *sp. Y1 and commercial protease, and only 24% were removed by glycine-NaOH buffer after degumming with the control pectinase, because it was equal to the control experiment with only glycine-NaOH buffer. This corresponded to the protease activity in the crude enzyme of *Bacillus *sp. Y1 (38.6 U/mL) and control pectinase (0 U/mL) (Table 1). But the increased gum loss was much higher than the effect of protein removal on weight loss. This significant difference of gum loss between the crude enzyme of *Bacillus *sp. Y1 and control pectinase (Figure 1) should attribute to not only the protein removal but also the promotion effect of protease on pectinase in degumming with the crude enzyme of *Bacillus *sp. Y1.

This suppose has been substantiated by measuring the concentration of released galacturonic acid in degumming liquid using the absorbance of unsaturated bonds at 235 nm [11] and reducing groups at 276 nm [24]. Kapoor et al. also used the determination of the concentration of released galacturonic acid to indicate the degumming efficiency [5]. A previous study also found that there was a good correlation between the gum loss and the released galacturonic acid measured at 235 nm and 276 nm (data not shown). Samples with no ramie fibers as the substrate were used as control. The results showed that the concentration of removed polygalacturonic acid in degumming liquid of degumming with mixture of control pectinase and commercial protease increased by 26% and 24%, respectively, meaning that the removed of polygalacturonic acid from ramie increased more than 24%, compared to with the control pectinase alone. Kirby et al. have reported that pectin extracts contain a mixture of pectin polysaccharides and protein-pectin complexes, and these protein-pectin complexes consist of pectin molecules with protein attached to one end of the pectin chain [25]. So, the protease should remove the protein in the pectin-protein complexes, which helps induce a reaction between pectin and pectinase, and leads to more pectin degradation and removal. 

To further substantiate the effect of protease on ramie degumming, the protease and PGL were purified (Figure 6), and the comparative degumming experiments with them were carried out. The concentration of released galacturonic acid in degumming liquor that measured at 235 nm and 276 nm increased by 44% and 64%, respectively, if using a mixture of purified PGL and purified protease instead of the purified PGL alone. These significant increments in removed polygalacturonic acid further substantiated the significant effect of protease on ramie degumming.

SEM observation was also used for studying the change of fibers before and after degumming with the purified protein, and shown in Figure 7. After degumming with purified PGL (Figures 7(b) and 7(e)) or mixture of purified PGL and purified protease (Figures 7(c) and 7(f)), the fibers were separated and the surface of fibers was much smoother with the removal of the encrusting materials and almost no cell wall debris remaining compared with the untreated (Figures 7(a) and 7(d)) and chemically degummed fibers [5]. However, application of mixture of purified PGL and purified protease produced fibers with an even smoother surface (Figures 7(c) and 7(f)), a complete removal of gummy material, than treated with the purified PGL alone (Figures 7(b) and 7(e)) and chemical and subsequently pectinase [5]. Furthermore, separation of the fibers was also significantly improved with mixture of purified PGL and purified protease compared to with the purified PGL alone. These appearances provide another strongly visible evidence for the important role of protease in degumming. The results provide new insights into the genetic modification of degumming strain and improvement of degumming enzyme system.

## 5. Conclusion

The crude enzyme of *Bacillus* sp. Y1 was found to have a powerful and fast degumming ability on ramie by screening wild-type strains. It mainly consisted of pectinase and protease from the active component of the enzyme. And its alkaline pectinase had suitable characteristics for degumming process, especially the high tolerance for H_2_O_2_. What is more, the gum loss and brightness of ramie fibers could be significantly improved when different concentration of H_2_O_2_ was added into it for degumming. The synergistic action was also found between it and H_2_O_2_ on the degumming and bleaching of ramie fibers. All showed that it was very suitable for combining enzymatic degumming and H_2_O_2_ bleaching, which could reduce process and devices costs. It also contained more proteins compared with the control pectinase, and protease component in it was further substantiated to play an important role in the degumming process. There was also a synergistic action between protease and pectinase on degumming. We believe that these results are very valuable for boosting the study of degumming enzymes, degumming mechanism, and application of enzymatic degumming technology in the industry.

## Figures and Tables

**Table 1:** Activities of enzymes.

Enzyme	Enzyme activities (U/mL)					
	PGL	PG	Xylanase	Mannase	FPase	Protease
Crude enzyme of strain Y1	26.99	39.97	1.22	0.76	0.00	38.60
Control pectinase	288.70	337.19	0.43	0.32	0.00	0.00
Protease	0.00	0.00	0.00	0.00	0.00	2037.40
Xylanase	0.00	0.00	22.58	0.00	0.00	0.00
Mannase	0.00	0.00	0.00	89.79	0.00	0.00
